# Challenges and Recent Progress in Oral Drug Delivery Systems for Biopharmaceuticals

**DOI:** 10.3390/pharmaceutics11030129

**Published:** 2019-03-19

**Authors:** Bahman Homayun, Xueting Lin, Hyo-Jick Choi

**Affiliations:** Department of Chemical and Materials Engineering, University of Alberta, Edmonton, AB T6G 1H9, Canada; homayun@ualberta.ca (B.H.); xueting2@ualberta.ca (X.L.)

**Keywords:** oral delivery, biological barriers, co-delivery, throughput, sustained delivery

## Abstract

Routes of drug administration and the corresponding physicochemical characteristics of a given route play significant roles in therapeutic efficacy and short term/long term biological effects. Each delivery method has favorable aspects and limitations, each requiring a specific delivery vehicles design. Among various routes, oral delivery has been recognized as the most attractive method, mainly due to its potential for solid formulations with long shelf life, sustained delivery, ease of administration and intensified immune response. At the same time, a few challenges exist in oral delivery, which have been the main research focus in the field in the past few years. The present work concisely reviews different administration routes as well as the advantages and disadvantages of each method, highlighting why oral delivery is currently the most promising approach. Subsequently, the present work discusses the main obstacles for oral systems and explains the most recent solutions proposed to deal with each issue.

## 1. Introduction

Intravenous (IV), intramuscular (IM), intranasal (IN), intradermal (ID)/transdermal and oral administration are the main drug delivery routes. Other routes, such as ocular delivery, have also been developed for localized, site-specific drug administration without unwanted systemic side effects [1]. Each administration method faces specific barriers against the delivery of the drugs. In addition, drugs can be incorporated into delivery devices, which considerably contribute to preservation of the drug, targeting and therapeutic efficacy. In this review, we first present an overview of the various administration routes, then focus on oral delivery systems as the most attractive route. We explain the main challenges associated with such methods and review the most recent solutions developed to address them.

The absorption mechanism as well as the nature of the drug are the fundamental factors that determine the appropriate delivery systems for achieving the highest bioavailability and effectivity. For instance, IM and ID administration are usually the preferred vaccination routes, depending upon the desired immune response mechanisms. On the other hand, researchers from both academia and industry have shown great interest in IN and oral vaccination systems, since these routes can induce both systemic and mucosal immune responses. In IV administration, the drug is rapidly injected into blood vessels through needles, and a high concentration of the drug is able to bypass the physiological barriers against drug absorption, providing the highest bioavailability and the fastest effect among all delivery routes. Therefore, such a parenteral administration is the preferred route for acute and emergency responses, while non-invasive methods are more suitable for sustained therapy and chronic delivery [2]. The abundance of blood vessels in muscles paves the way for the absorption of drugs injected via needles through IM administration. IM delivery bypasses the body’s first defense barrier (skin) [3]. In comparison with oral administration, drugs administered through the IM route avoid the gastrointestinal (GI) environment. However, the injection can cause significant problems, including needle-associated phobia and pain, unsafe needle use and improper disposal, the need for trained healthcare personnel, muscle atrophy, and injuries to bones and nerves [4]. Additionally, there is concern over the direct injection of drugs into the bloodstream through IM administration, necessitating constant close observation to minimize adverse effects [5,6].

Biopharmaceuticals such as vaccines are of particular interest in drug delivery because of their specific challenges. The majority of the available vaccines are administered through IM injection [7]. This is mainly due to the poor permeability of macromolecular biopharmaceuticals across the mucosal layer in the non-parenteral route and the destructive effects of proteases in the GI tract [8]. Silica and polymer mesoporous structures can also be successfully used to preserve drugs in various biological surroundings and accurately control their release behavior in topical injections [9,10,11]. However, it should be noted that IM administration is not the ideal delivery route for peptides and proteins, compared to subcutaneous or IV injection, mainly because of the low immunogenicity and bioavailability achieved in IM administration [12]. Although IM vaccination is widely used commercially and the immune response in this system can be easily induced by the local depot at the injection site, this route is not the best choice for the delivery of peptides/proteins due to the possible aggregation of the drug [13]. 

The transdermal route concerns the delivery of drugs across skin layers to the blood circulatory system [14]. Drug absorption in this case mainly occurs through the intercellular, transcellular and transappendageal pathways. Intercellular and transcellular transports enable the permeation through the stratum corneum [15]. In the transappendageal pathway, the drug penetrates via the sweat ducts or the hair follicles with their associated sebaceous glands [14]. The transdermal route also avoids the challenges that the oral route faces, including the metabolism and the difficulties associated with the GI environment. Moreover, it can provide a sustained drug plasma level, and convenience of discontinuation of the drug in case adverse reactions occur. Recently, nanoparticles (NPs) were successfully employed on nano/micro-engineered needle patches to minimize the bacterial risks associated with transdermal delivery techniques [16]. Nanoimprint lithography has also been proposed as a fabrication technique for these structures to address the commercial requirements. Kim et al. also proposed a new deposition-etching protocol for the fabrication of flexible silicon nanoneedles to solve the structural mismatch and optical inconsistency between the Si needle wafers and soft tissues [17]. The patches fabricated by this technique exhibited successful injection of biomolecules into living tissues. Nonetheless, the most significant challenge associated with transdermal administration is the restriction over the size of the drug molecules that can be successfully delivered. Penetration of large molecules (>500 Da) through the stratum corneum is difficult. Additionally, the drugs are required to be soluble, so they can cross the outermost skin barrier [18]. Chemical enhancers (concerning the delivery of small-sized molecules) and physical approaches (for the delivery of macromolecules) have been developed to improve drug absorption levels in the transdermal delivery route. Physical skin permeability enhancement regards electrically-assisted methods such as electroporation, iontophoresis and sonophoresis. Chemical enhancers include fatty acids, surfactants, terpenes and solvents, which improve skin permeability by disrupting the highly ordered lipids and modifying the stratum corneum microstructure [19,20]. However, toxicity and skin irritation are the major concerns to consider when developing chemical enhancer formulations. Karande et al. used a library of more than 4000 binary formulations to systematically investigate the synergistic effects of different enhancers, and found some fundamental rules in terms of developing new formulations [21]. While these rules are well-known and widely accepted, the mechanisms behind such potential synergistic effects and the interactions each individual chemical enhancer may have with other enhancers or the stratum corneum still remain unclear.

Medications can sometimes be injected directly into veins. This is known as intravenous administration. In this scheme, the drug directly enters the circulatory system without facing any physical, chemical or biological barriers. Since the absorption of the drug is guaranteed and immediate, IV administration is the best route for emergency situations. Additionally, this method uniquely provides a very precise control over the dosage and speed of administration, making it the best approach for drugs requiring a stringent dosage [22]. On the other hand, the IV route entails risks of injury, infection by the needle at the site of injection, circulatory overload, phlebitis and thrombosis [23,24]. IV administration is commonly used for the delivery of biopharmaceuticals. The bioavailability of the drug injected through IV route is theoretically 100%, making this system outperform other delivery routes. However, it should be noted that IV administration is not the ideal route for the delivery of vaccines. This is due to the difficulties associated with inducing effective immune responses via IV administration, since the IV route does not provide an adequate local depot of antigens to stimulate/activate the innate immune response and induce the long-term secretion of antibodies [25]. In addition, it is not applicable to implement a mass administration (such as nation-wide vaccinations), due to the skills required for the practitioner, safety issues and patients’ compliance. 

Intranasal drug delivery entails the infusion of the drug into the highly vascularized mucosal layer of the nose to subsequently reach systemic circulation [26]. IN drug delivery is crucially significant for neurological diseases, where drugs are required to reach the central nervous system (CNS) by bypassing the blood-brain barrier (BBB). In general, the IN route is preferred for local diseases due to its limited systemic effects compared to the other methods. The IN route also has its own specific physiological and physicochemical barriers. The physiological barriers include capillary barriers, nasal mucus, mucus clearance and nasal metabolism. Other factors such as pH, possible drug-mucus interactions and the viscosity of the mucus may also influence drug diffusion and absorption in IN administration. Mucoadhesive microencapsulation systems have been developed to deal with the mucus-associated barriers of IN delivery and improve the bioavailability of nasally-administered drugs. For example, Nanaki et al. coated nasal microcapsules with thiolated chitosan to aid the mucoadhesion of the system, both physically (electrostatic attractions) and chemically (disulfide bonds) [27]. However, it should be noted that mucociliary clearance and discharge considerably limits the drug residence time, even if the carriers develop a strong bonding/binding with the mucus [28]. Physicochemical barriers concern the molecular weight of the drug, and its lipophilicity and degree of ionization, which also define the absorption mechanisms [29]. Despite multiple challenges, IN is an advantageous route for the delivery of a variety of the drugs. It avoids first-pass metabolism and GI complications. Due to its considerable absorption rate, IN is an applicable method for emergency cases and rapid drug action. Neurological drugs especially can be transported directly to the CNS through the IN route.

The nasal-associated lymphoid tissue (NALT) is the principal target for inducing mucosal immunity in nasal vaccination. The innate immunity is achieved by macrophages and dendritic cells, and the adaptive immunity at the mucosal layer is induced by IgA [30]. Hence, IN vaccination can induce both mucosal and systemic immunity. However, physiological barriers, especially mucociliary clearance, need to be adequately addressed for the effective delivery of antigens to the target site. Furthermore, due to the limitations of the nasal cavity, as well as the narrow passages beneath the thick mucus, nasal vaccination is only permitted for small dosage and low molecular weight compounds. In order to pass biosafety requirements, IN delivery devices need to be suitable for narrow nasal entrances and the complex geometry of the nasal passage. On top of that, lung exposure should be properly addressed in these systems. Among the various physical states of drugs (gels, droplets, powders, or aerosol sprays), aerosol sprays are usually preferred for IN administration. New technologies are constantly under development to improve the dispersion of the drugs and modify the deposition and clearance behavior through combining solid and liquid phases. However, human anatomy as well as the physiology of the nasal cavity and passage still limit clinical applications and delivery efficiency. Low bioavailability (<5%) causes another major challenge for IN administration systems [31].

## 2. Oral Route

Among the various drug delivery routes, the oral pathway has attracted the most attention due to its unique advantages, including sustained and controllable delivery, ease of administration, feasibility for solid formulations, patient compliance and an intensified immune response in the case of vaccines [32,33,34,35,36]. In addition, a large surface area (>300 m^2^) lined with a viscous mucosal layer paves the way for drug attachment and subsequent absorption [37,38]. Furthermore, drug molecules trapped within mucus are protected against the shear stresses caused by flowing gastric juices [39]. The epithelium of the human intestine is very absorptive due to the abundance of enterocytes in different parts of the intestine, especially microfold cells (M cells) covering the Peyer’s patches, the lymphoid segment of the small intestine [40,41,42,43,44]. However, in comparison with other routes, the absorption mechanism of oral drugs is more complex. Oral drugs need to be soluble in gastric fluid so they can be absorbed in the stomach, the small intestine or the colon (Table 1). Orally administered drugs can be absorbed in four types of pathways: Transcellular, paracellular, carrier-mediated transcellular and facilitated transport. Among these pathways, the transcellular pathway is the main mechanism. The challenges of drug absorption/efficacy do not limit the barriers met in the gut, but they include the hepatic barriers after they enter the vessels under the intestinal epithelium as well. In summary, oral drugs are not applicable for emergencies due to their slow absorption as well as the multiple levels of barriers they need to deal with.

Although the oral route is the most desirable administration method for small therapeutic molecules, there are not so many oral vaccines on the market due to the harsh conditions along the GI tract (Table 1), which can degrade/denature active antigens. 

However, the attraction of the mucosal immunity, which appears to be induced by oral and nasal routes, promotes the study of oral vaccines [49]. Besides, the convenience and other advantages of oral delivery make it a very promising strategy for mass vaccination programs. The inductive sites in the GI tract consist of Peyer’s patches, lymphoid follicles in lymph nodes and antigen presenting cells (APCs). Intestine mucosal immunity is similar to that of nasal mucosal immunity. The main barrier for vaccine delivery is the change of pH in different sites in the GI tract and various enzymes, making it hard to permeate the mucus and reach the inductive site in gut-associated lymphoid tissue (GALT) [50]. Additionally, the mucosa may lead to the structural change of proteins and peptides due to various possible interactions [38]. Hence, delivery vehicles and formulations should be developed to gain a stronger immunogenicity to meet the required therapeutic efficacy. Currently, seven live oral vaccines have already been approved by FDA. 

To meet the increasing demand for biopharmaceutical oral products, research has been focused on developing devices for oral delivery. While still at an early stage, recent devices include intestinal patch systems, microneedle capsules and particulate systems [51]. The intestinal patch systems are based on a unidirectional drug release depot, which is similar to a microdevice adhered to the intestinal wall [52]. The microneedle capsule increases the penetration rate of drug molecules by piercing the mucosa directly with microneedles. A recent study developed a method to inflate a microneedle into the mucosa by responding to the change in pH [53]. Particulate devices are the most common oral vehicles, which have been investigated for the encapsulation and targeting of a vast variety of therapeutics. In general, the current technologies are still at the preclinical stage. Therefore, more research efforts should be directed to solve the existing technical challenges of oral drug delivery systems and prove the feasibility in clinical use.

## 3. Challenges Associated with Oral Delivery

Oral drugs are transported and absorbed in the GI tract, which is in the shape of a conduit. Some drugs have local effects in the gut, while most of them are sent to the bloodstream in the systemic circulation to act in other parts of the body. The GI tract can be divided into upper and lower parts. The upper GI tract includes the oral cavity, pharynx, esophagus, stomach and the initial part of the small intestine, known as the duodenum. The lower GI tract includes the rest of the small intestine (jejunum and ileum), as well as the large intestine segments: The cecum, colon and rectum [54,55]. The structure of the GI tract is similar in all segments. The lumen is enveloped by smooth muscle cells, covered by mucus, submucosa and several muscle layers [56]. The mucosal layer which lines the inner part of the GI tract consists of a layer of epithelial cells, lamina propria and muscularis mucosae, which play significant roles in food/drug molecule transport and gastrointestinal immunity [54,55]. A large absorption area and long residence time provides greater opportunities for drug absorption, which is one of the reasons why drug absorption mostly occurs in the small intestine. Further, between the three main parts of the small intestine (duodenum, jejunum and ileum), the jejunum and ileum have a higher absorption capability compared to the duodenum [57,58]. 

The environmental factors that influence drug integrity and absorption include the average length of the segment, pH, thickness of the mucus, residence time of the drug and the bacterial diversity/population in different segments [38,59,60]. The obstacles against oral administration may be broadly classified into biological barriers and technical challenges. Biological barriers include any biological factors that denature the orally administered drugs or prevent their successful absorption in the target. On the other hand, technical challenges relate to any difficulty in the fabrication process of the oral delivery devices. The technical challenges may either be issues with creating specific properties for addressing the biological barriers or complications with scaling up and commercializing a system. The details of each category will be discussed in the following sections.

### 3.1. Biological Barriers

Any digested ingredient will be dealing with three main biological environments along the gastrointestinal (GI) tract, regardless of its absorption mechanism or target. These environments are the lumen (i.e., the interior space), mucus and tissue. Each of these three environments may have interactions with the drug molecules.

#### 3.1.1. Lumen

The first biological barrier against any orally administered drug is the harsh acidic conditions inside the stomach (pH 1–2.5), denaturing/depurinating most of the administered molecules, drastically lowering their effectivity [61,62,63,64]. In addition to stomach acid, gastric enzymes such as pepsin and gelatinase can degrade biopharmaceuticals. pH-responsive hydrogels can encapsulate the drugs and protect them not only against the harsh acidic environment, but against gastric enzymes as well. These materials can remain intact in unfavorable surrounding conditions to protect the loaded drug (promptly reacting to environmental stimuli such as the pH in the target) and release the cargo. For instance, Yamagata et al. confirmed that pH-sensitive hydrogel microparticles (MPs) can efficiently preserve sensitive drugs such as insulin against gastric/intestinal enzyme fluids [65]. In another study, Cerchiara et al. also developed an oral pH-responsive microencapsulation system and demonstrated its capability for protection against both gastric acidic and gastric enzymatic environments [66].

Notably, in addition to the gastric enzymes, there are also pancreatic enzymes synthesized inside the pancreas and secreted into the intestinal lumen. These enzymes include lipase (degrading fats), trypsin (decomposing proteins), amylase (degrading starch) and peptidases (disintegrating peptides), and are especially abundant at the main entrance of the small intestine (duodenum). They can readily decompose nucleic acids and reduce the gastric residential stability of biomolecules [67,68]. Although pancreatic enzymes are also introduced as biological barriers against oral delivery, they are not considered as a major challenge due to three principal reasons. First, these enzymes are mainly abundant in the duodenum, and their concentrations considerably decrease in the jejunum and later parts [69]. In addition, Layer et al. attempted to deliberately deliver pancreatic enzymes to the intestine and noticed that even the concentration of the delivered enzymes substantially decreases in the midjejunum compared to that in the duodenum (they reported up to a 90% drop in enzymatic activities) [70]. The second aspect is the short transit time of the digested food inside the duodenum (Table 1), which is not enough for the enzymes to degrade the drugs. Fallingborg et al. reported that duodenal residence comprises only 10% of the whole small intestine average residence time [71]. Lastly, the pH of the duodenum is lower than that of the later parts of the lower small intestine (Table 1), meaning that unwanted release of the drugs inside the duodenum can be successfully avoided by controlling (increasing) the pKa of the delivery carriers. For example, Lozoya-Agullo et al. employed poly(lactic-*co*-glycolic)acid (PLGA) nanoparticles for colon delivery and confirmed that duodenal release may be significantly avoided due to the insufficient environmental pH [72].

In addition to the acidic and enzymatic degradation, the lumen can cause other damages to drug molecules. Osmotic stresses along the GI tract, peristalsis of the GI muscles, as well as the shear stresses by the flow rate of the gastric juice inside the lumen are other factors that decrease drug efficiency due to mechanical degradation inside the lumen [32,38]. Flowing gastric juice may also decrease the contact time between the drug molecules and the epithelial layer, thus impeding their absorption [67]. Enveloped biologics such as viruses, vaccines and cells are usually the main components sensitive to mechanical destruction. Valon et al. studied the possible effects of mechanical stresses on various types of cells, and reported that shear stresses and compaction may lead to apoptosis and cell death [73]. Choi et al. also found that hyperosmotic pressure can destroy virus integrity in an acidic environment [32]. Although mechanical stresses by the lumen can destroy biological agents, microencapsulation can properly address these problems as well. Indeed, MPs are hardly studied for mechanical testing, and mechanical strength is usually discussed in regard to hydrogel properties, which in turn is a function of the molecular weight of the monomers as well as the degree of crosslinking of the matrix. Nanoparticles may also be added to hydrogels as reinforcing components. For instance, France et al. recently employed cellulose nanocrystals (CNCs) to create physical crosslinking in a hydrogel matrix, proving their significant impact on mechanical behavior (up to a 35-fold increase in the shear storage modulus of the final composite) [74]. Yang et al. also reported up to a 3.5-fold improvement in the Young’s modulus of poly(ethylene glycol) (PEG) hydrogels through the addition of 1.2 vol % CNCs [75]. As an instance of ceramic-polymer composite systems, Gaharwar et al. used hydroxyapatite as the reinforcing agent in a ceramic-polymer composite and reported up to a 1.7-fold improvement in the compressive strength of the matrix. It should be noted that the addition of secondary components to form a composite will not only affect the mechanical properties, but may also affect the swelling behavior, gelation rate and degradation rate of the hydrogels as well [74,75]. Furthermore, the distribution mechanisms of CNCs throughout the polymer matrix are poorly understood, and there are underwhelming (sometimes contradictory) reports about the improvements in mechanical properties of composites. However, the greatest and most effective improvement reported by the formation of a hydrogel composite to date concerns the addition of CNCs to hydrogels, without the need for any surface or other secondary modification of the particles.

#### 3.1.2. Mucus

Mucus is the second compartment of the GI tract, which any digested moiety interacts with. The entire GI tract is lined by mucus (a sticky, elastic and viscous layer), responsible for the capture of foreign moieties, especially hydrophobic molecules, impeding their contact with the underlying epithelial layer. The mucus then discards such foreign moieties, acting as one of the main compartments of the immune system, known as mucosal immunity [76,77,78,79]. The mucus itself is mainly composed of water and mucin protein molecules coated with proteoglycans, giving the mucus a negative charge [80]. Carbohydrates, salts, bacteria, antibodies and cellular remnants are the other compounds found in mucus [81]. The thickness of this layer is reported to vary along the GI tract, as summarized in Table 1 [38]. 

**Figure 1:**
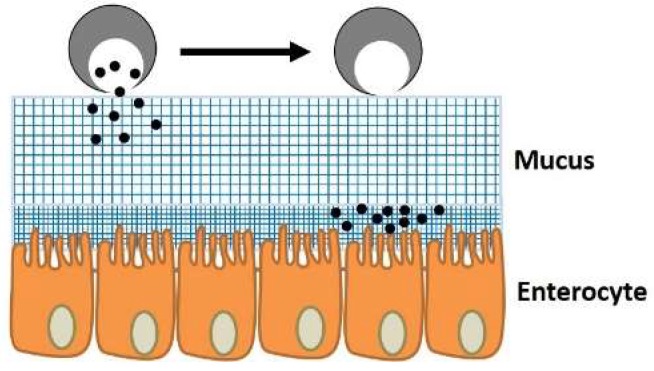
The structure and function of the mucus. The schematic shows the gastric mucus layer, the attachment of particles to the outer and inner mucus, and the drug delivery vehicle on the outer mucus.

Mucins are large macromolecules (0.5–40 MDa) made of monomers connected through disulfide bonding, and these macromolecules are subsequently crosslinked to build up the mucosal layer [82,83]. The mucosal layer itself is usually composed of two separate overlaying layers: An outer loosely adherent layer and an inner firmly adherent layer (Figure 1) [84]. The inner layer is composed of glycoproteins, glycolipids and cell-bound mucin [85,86]. It has been claimed that the thicker outer mucus acts as a barrier against the transition of released drugs to the submucosal tissue. For instance, Marie Boegh and Hanne Mørck Nielsen studied the diffusion of peptides and proteins through mucus and found that mucus is the main obstacle against the bioavailability of oral drugs [87]. On the other hand, the narrower inner mucus is known to help the absorption/enhance the uptake efficiency of drugs, justifying the dual role of mucus in the absorption/desorption of orally delivered drugs (Figure 1) [88,89]. There is an equilibrium between mucin secretion, degradation and clearance in each segment of the GI tract to protect the epithelium and control the nutrition absorption rate, which in turn defines the final thickness of the regional mucosal layer (Table 1) [38,90].

The approaches taken regarding mucus in the field of oral delivery can be generally classified into two opposite mainstreams: Mucopenetration and mucoadhesion. Mucopenetrating oral vehicles may be made through controlling the hydrophobicity/hydrophilicity nature of the carriers’ matrix. In other words, due to the hydrophobic nature of the outer mucus, it tends to have significantly less interactions with hydrophilic materials. Additionally, regulating the electrostatic interactions between the carriers and the mucus can dramatically facilitate the transition of particles through mucus. Li et al. incorporated Pluronic F127 (PF127) into the matrix of liposomes to induce hydrophilicity and nullify the electrostatic charge of the particles, demonstrating considerable improvements in the mucopenetration and uptake efficiency of the liposomes [91]. Cu et al. reported similar improvements in terms of the mucopenetration of poly(lactic-*co*-glycolic)acid (PLGA) nanoparticles by coating them using PEG, which neutralized the surface charge of the nanoparticles [92]. 

The other platform employed for penetrating through mucus is the application of mucolytic enzymes. Muller et al. fabricated nanoparticles made of complexes of poly(acrylic acid) (PAA) and papain (a mucolytic enzyme) through physical adsorption [93]. They showed that the enzyme-conjugated particles can reduce the viscosity of fresh mucus by up to 5 times, reflecting the potential of the system for improved oral bioavailability. Pereira de Sousa implemented a similar study comparing bromelain (another mucolytic enzyme) with papain for the same purpose [94]. They showed that the bromelain-modified particles exhibited a significantly enhanced mucopenetration behavior compared to the papain-conjugated particles (up to a 4-fold increase in mucopenetration compared to the papain-conjugated particles, and up to a 10-fold increase compared to the blank samples). 

On the other hand, the protective and sticky properties of mucus can be exploited to protect the digested ingredients and extend their transition time along the GI tract. As a result, mucoadhesive drug carriers have attracted significant attention [95,96]. For this purpose, cationic hydrogels such as chitosan have been extensively investigated [97,98,99]. Recently, Kim et al. derived catechols from mussels and conjugated it with chitosan to develop a new complex with significantly better mucoadhesive properties and negligible cytotoxicity compared to chitosan alone [100]. Lectin functionalization has been another strategy employed for the same purpose. The study implemented by Ertl et al. is one of the first works with this approach, in which they conjugated wheat germ agglutinin with PLGA MPs and increased their mucoadhesion [101]. The advantage of this new system is that lectin-conjugated MPs exhibited improved adhesion not only to the mucus but also to the enterocyte (cell) surfaces, minimizing the problems associated with the short turnover period of mucus. Additionally, in specific parts of the GI tract where the mucus is not thick enough, this system can still show improved absorption efficiency.

In the case of anionic MPs, their surfaces have been functionalized with thiol functional groups to exhibit mucoadhesive properties by forming disulfide bonds with thiol groups existing in mucins [102,103,104,105,106,107]. Several studies have confirmed that such chemically-modified MPs show a significantly longer residence time in the target, acting as potential candidates for the sustained oral delivery of specific drugs, such as insulin and losartan [96,108]. For instance, Zhang et al. functionalized Eudragit L100 with thiol groups and confirmed improvement in the absorption and bioavailability of orally administered insulin compared to the unmodified polymer by monitoring the blood glucose concentration [108]. However, it should be noted that mucus is constantly secreted by goblet cells along the GI tract and is subsequently shed and cleared from tissues due to the rapid turnover of cells. This leads to a very short residence time for the attached agents to reach the epithelium for absorption (50–270 min) [109,110]. Mucus, with around a two day turnover period, plays the most significant role as a barrier [111,112]. Hence, sustained delivery using mucoadhesive particles may not represent the ideal strategy in this regard. In addition to this, it is difficult to model the real effects of mucus on delivery carriers in vitro due to its changing thickness along the GI tract and the constant effects of flowing gastric juice [38,67,112]. Since sustained oral delivery itself is a major challenge, it will be discussed under a separate subsection later in this review.

#### 3.1.3. Tissue (Extracellular Barriers)

**Figure 2:**
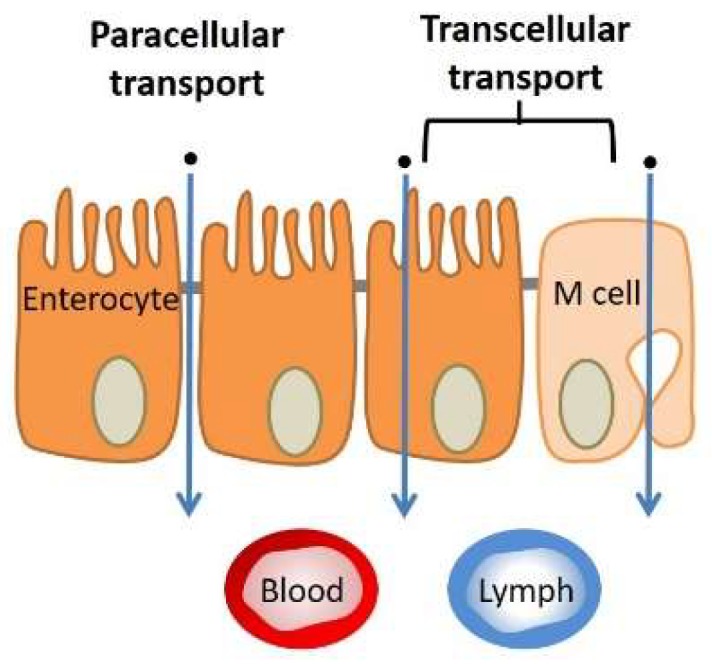
Absorption mechanisms through the mucosal layer. Paracellular route to lamina propia and transcellular route (enterocytes, M-cells, transfection of the epithelial cells, direct absorption through dendritic cells and active transport).

The characteristics of the drug molecule will determine its absorption sites and its pathway to cross through the intestinal epithelial cells in the GI tract. There are two main pathways for the absorption of oral MPs or drugs: Transcytosis by cells (transcellular route) and diffusion through the spaces between epithelial cells (paracellular route). Figure 2 schematically represents all the possible absorption scenarios that a digested molecule may encounter in the intestinal lumen. In the transcellular pathway, the drug molecules enter the enterocytes by crossing the membrane of the epithelial cells. The paracellular pathway permits only small hydrophilic molecules to be absorbed, playing a minor role in drug absorption as it is narrow and only occupies a small area fraction of the whole epithelium [87]. The principal extracellular biological barrier against oral delivery is known as tight junctions, which concern the paracellular absorption route for orally administered agents [113,114]. 

The transport of MPs or drug molecules through tissue depends on both their chemistry and their size. Generally, hydrophobic drug molecules, nanoparticles, vesicles and micelles prefer to be absorbed through transcellular routes due to their large size and chemistry, while hydrophilic small drug molecules prefer paracellular routes [67,115]. There are also barriers due to the structure of the cells. At the outer regions of the membrane bilayers, the high molecular density of the polar head groups of the lipid membranes makes it difficult for the drug molecules to pass through the cell membranes. Furthermore, after entering the cell membrane, cellular components such as enzymes may degrade/decompose the drug molecules in the cytosol, decreasing the therapeutic efficiency [89].

Passive diffusion of MPs through intercellular routes is only possible for agents with sizes of up to a few nanometers (0.5–3 nm), which is too small for the delivery of most drug molecules [116,117]. A notable example of this problem is the poor bioavailability of doxorubicin (DOX), which is attributed to its limited paracellular absorption in the intestine. Kim et al. developed a medium-sized chain glyceride-based water-in-oil (W/O) microemulsion system to overcome the paracellular barrier against DOX intestinal absorption [118]. The improvements in the absorption level of the drug were ascribed to the lipidic components of the carriers, inducing the paracellular enhancing effects. In addition to the chemistry of the carriers, there are few absorption enhancing agents that have been used in oral delivery systems. Sodium *N*-[8 (2-hydroxylbenzoyl) amino] caprylate (SNAC) is a paracellular permeability enhancer which has been recently used for clinical trials by Davies et al. [69]. The problem with this agent is that there is no distinct mechanism identified for its function. Recently, Taverner et al. identified other peptides (PIP peptides: 250 and 640), which could enhance the paracellular permeability of insulin through intestinal tissue [119]. They claimed that these peptides can dynamically adjust endogenous mechanisms, inducing myosin light chains (MLCs), opening the tight junctions and facilitating paracellular transition, especially for peptide therapeutics. Almansour et al. (in the same group) studied PIP 640 further and confirmed its stability in the intestinal lumen environment and explained its functioning mechanism: PIP 640 selectively enhances the MLC-pS^19^ levels of the cytoplasm of enterocytes in the epithelial layer [120]. Apart from the recent studies and the progress made, tight junctions are still one of the main challenges against the absorption of biopharmaceuticals. The lack of a fundamental understanding of the mechanisms controlling tissue permeability and the effects of various agents may be one of the principal reasons for this problem.

### 3.2. Technical Challenges

In addition to biological barriers, oral delivery systems face technical difficulties as well, in terms of deciding whether to induce new properties addressing biological barriers or to scale up existing systems for commercial purposes. In this section, most common oral delivery devices, sustained delivery strategies, solvent-free microencapsulation techniques, co-delivery systems and the challenges associated with the scaling-up of systems are analyzed. 

#### 3.2.1. Oral Delivery Devices and Materials

**Figure 3:**
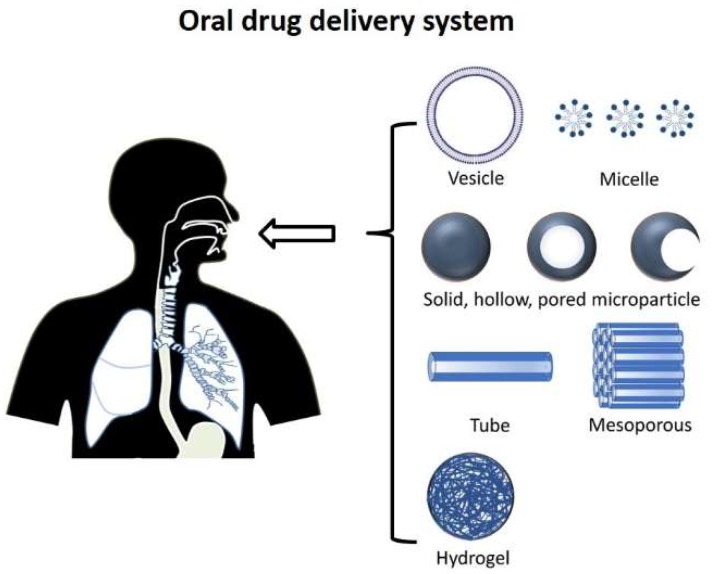
Schematic illustration of the principle oral vehicles designs.

The devices developed for oral drug administration may be classified as intestinal patches, gastrointestinal microneedles and particulate carriers (including micro/nanoparticles, micelles and liposomes), as illustrated in Figure 3. Intestinal patches are millimeter-sized mucoadhesive blankets that attach to the inner walls of the GI tract, providing a drug reservoir at the target. These patches can protect the drug against the harsh environment and luminal loss, improving the bioavailability of the drug by providing a unidirectional diffusion regime towards the intestinal tissue. They have been especially attractive for improving the oral bioavailability of drugs and sustained delivery. Insulin, interferon-α and calcitonin are examples of drugs investigated for delivery using such devices [121]. As far as intestinal patches are concerned, the mucosal adhesion properties, loading capacity, release rate and release direction are the main factors to be considered. Mitragotri et al. have used mucoadhesive polymers, such as Eudragit copolymers or pectin, to prolong the gastric residence time of devices [122]. They also used impermeable ethyl cellulose sheets to create a unidirectional release pattern and seal the opposite side of the patches. Shen et al. also showed that incorporation of drug-loaded microspheres into the patches, instead of direct loading of the drugs, can provide significantly enhanced control over the release behavior of the drug [123]. Toorisaka et al. developed a lipophilic formulation (drug-in-oil formulation) to improve the compatibility of the system with intestinal cell lines and enhance the absorption of insulin [124]. However, their formulation lacked enough retention time at the target. They later solved this issue by designing a bilayer patch consisting of a drug-impermeable layer to guarantee a unidirectional drug release regime and a mucoadhesive layer to prolong the gastro-residence time [125]. The drug-in-oil formulation was also impregnated into the porous mucoadhesive layer. Although it has been more than two decades since the development of these oral devices, they have not attracted as much research attention as other designs, such as MPs. Oral patches are mainly applicable in the initial segments of the duodenum, since solid boluses of digested food can detach the patch from the lumen wall in later parts of the GI tract, significantly decreasing the transient time. Even in the duodenal part, the device needs strong bonding/binding with the mucus to avoid being washed away by gastric juice. Furthermore, the mucus turnover cycle limits the real application of these devices for sustained delivery. 

Intestinal microneedles are the newest design developed as an oral delivery device. Microneedles were first developed for transdermal delivery (transdermal patches), and their application was subsequently extended to other administration routes including the vagina, anus and scalp [126]. In 2014, Ma et al. employed microneedles for oral vaccine delivery to the mouth cavity, which was the first time microneedles were used for oral administration [127]. They investigated the system for the delivery of two HIV antigens (DNA vaccines and virus-like particles), comparing the induced immune response with the response generated by the intramuscular injection of the drugs. They reported that only orally administered agents showed a stimulated antigen-specific IgA response in saliva. The limitation of this design is that it could only be employed for delivery to the oral cavity rather than the GI tract. Traverso et al. in 2015 tried to overcome the biological barriers of the GI tract using orally ingested microneedles and improved drug bioavailability [128]. They claimed that their device can be safely excreted from the GI tract. This design is especially promising for the delivery and successful absorption of large-size biomolecules. Their system demonstrated significant improvements in insulin bioavailability compared to the subcutaneous administration route. As a newly developed system, oral microneedles need to be studied considerably more, especially through clinical trials. There are currently not many investigations on this system.

Spherical carrier designs, including micelles, liposomes and MPs, are the most commonly studied oral delivery vehicles. Micelles are colloidal carriers (5–100 nm) developed to improve the aqueous solubility of hydrophobic pharmaceuticals and facilitate their oral delivery. They are made of amphiphilic molecules, enfolding the hydrophilic ingredients inside their hydrophobic core, with hydrophilic segments oriented on the outer walls. For instance, Dabholkar et al. developed a polymeric composition (polyethylene glycol-phosphatidylethanolamine conjugate) and increased the water solubility of paclitaxel (an anticancer drug) up to 5000 times [129]. Due to their very small average size (20–80 nm), micelles show spontaneous penetration through the interstitium of various tissues, improving their permeation efficiency and retention time. Yu et al. developed dual-responsive (pH and light) micelles to improve the passive tumor targeting of doxorubicin and address tumor resistance against the drug [130]. The micelles could trigger both deep tumor penetration and cytoplasm drug release, which in turn considerably improved treatment efficiency. Additionally, they found that the micelles could prolong the blood circulation cycle of the drug.

In addition to passive penetration, micelles can also help the active penetration of drugs. Suzuki et al. used cationic micelles (PLGA-*b*-bPEI-*b*-PLGA; Mw(PLGA): 36 kDa, Mw(bPEI): 25 kDa) for the encapsulation of doxorubicin and confirmed up to a 40-fold increase for in vitro drug penetration into multilayer cell cultures [131]. They claimed that iterative transcytosis via macropinocytosis and exocytosis are the main mechanisms for the penetration of the cationic micelles into cells. Although micelles are constantly proving their potential for improving therapeutic efficacy in bench-scale studies, they have hardly been commercialized. The clinical trials for these delivery devices are limited to a few cases for parenteral cancer therapies: Doxorubicin and its derivatives. A possible reason for this may be the safety of the materials, in terms of the physicochemical interactions between the carriers and mucus in real conditions [132]. On the other hand, liposomes are phospholipid vesicles (>200 nm) which can encapsulate both hydrophobic drugs in their hydrophobic compartment and hydrophilic drugs in their inner hydrophilic core. These carriers can be chemically modified through the immobilization of antibodies on their surfaces for improved target specificity. It should be noted that antibody-decorated liposomes may suffer from a short life cycle in blood circulation due to their accumulation in the liver, especially in the absence of sufficient target antigens [133].

MPs are the other common oral delivery architecture. The materials used in oral microencapsulation systems can be broadly classified into polymers and ceramics. Ceramics are usually safe materials for delivery applications due to their bio-inert nature. There are various ceramic materials used for delivery applications, including silica, alumina and calcium phosphate. For instance, cisplatin, methotrexate and hydrocortisone acetate have been successfully delivered using calcium phosphate carriers, and silica nanoparticles are dominantly used for chemotherapy [134]. Regarding the polymers employed for oral delivery, hydrogels are the most attractive structural materials, mainly due to their controllable chemical composition, tunable mechanical properties, water absorption, ability for internal material flow, and, above all, their capacity for stimuli-responsivity [135,136,137,138,139]. One of the primary features to control in hydrogels is porosity, since pore size can significantly affect the mechanical properties, uptake efficiency of extrinsic occupants, water/material flow through the polymer matrix and swelling ratio of the gel. The larger the mesh size, the easier the transport of materials through the structure is, and the higher the swelling ratio. For instance, Torres-Lugo et al. could regulate the release rate of salmon calcitonin from poly-(methacrylic acid) (PMAA) acid MPs by up to 50% by controlling the swelling ratio and mesh size of the hydrogel [140].

There are other strategies to increase the porosity of the hydrogel structure to a larger scale (micron scale) [141,142,143]. For instance, leaching out the template materials from the structure network is a common method for making porous materials [144]. Also, the number of crosslinking sites can be decreased through selective removal of one phase from the gel. Similarly, the aqueous/organic liquid absorbed inside the gel may be lyophilized, and the volume increase through the freezing process can create pores inside the polymer matrix [145,146,147]. The size of the pores created through this process depends upon the size of the ice crystals formed in the solid state and may range from nanoscale, by freezing the remaining molecular water contents inside the structure, to microscale macropores by concentrating the liquid at specific spots [148]. It should be noted that although removing crosslinking sites considerably affects the mechanical properties and the swelling ratio of the polymer, the porosity created by the frozen liquid does not change the hydrogel mesh size and consequently its properties/behavior [143]. 

In addition to achieving the optimum porosity for delivering small drug molecules, another major challenge is obtaining macropores for loading large biomolecules and cells into hydrogels [149,150]. If the pores are aligned in well-oriented geometries, the hydrogels can be used for directional cell growth and the creation of arrays [151]. Also, pore size and geometry define the nutrition transfer rate and cell migration pattern [152,153,154]. With all these aspects taken into account, developing methods for accurate control over the porosity of the hydrogel and size and morphology of pores in polymers has always been regarded as a main research topic for a range of various applications. France et al. recently reviewed the major methods developed for creating macroporous hydrogels [143]. 

#### 3.2.2. Sustained Delivery

##### Mucoadhesive Carriers

The maintenance of a constant concentration of the drug in the blood stream is a favorable situation for the great majority of treatment cases [155]. On the other hand, severe fluctuations in drug concentration can cause serious problems such as toxicity or ineffective treatment, reflecting the possible negative effects of burst release and the need for sustained delivery approaches [156]. Although sustained delivery can be barely achieved in some administration routes, like transdermal approaches, oral delivery is known as one of the potential candidates for this goal. Materials developed for sustained delivery are either positively charged, in order to attach to mucus through electrostatic binding (e.g., cationic hydrogels made from chitosan), or thiol-functionalized hydrogels, to attach to the mucin glycoprotein through disulfide bonding [157,158]. Although these approaches have shown higher levels of attachment to mucus compared to the control samples, these particles can only slightly increase the delivery time (in the range of a few hours), due to the short turnover cycle of mucus in the intestine [67,80]. Furthermore, MPs employed for oral delivery are usually made of pH-responsive polymers, releasing their cargo only at pH values above the pKa of the hydrogel, which is usually >6 for oral applications. Although the pH in most of the spots of the intestine is usually above 6 inside the lumen (Table 1), which in turn may significantly affect the ionization/swelling/dissolution of the MPs embedded in the mucus [159]. As such, there are in vivo studies that report intact MPs made of Eudragit L100/S100 leave the body without releasing their cargo. Notably, mucoadhesion is sometimes described as increasing the friction between the drugs or the delivery device with the GI tract walls [160,161]. This approach may also extend the delivery time, however, only in the order of few hours, which may still not be long enough for many cases [160].

##### Recent Gastric-Resident Architectures

Apart from the two approaches already discussed, Traverso and Langer proposed the idea to make delivery architectures larger than specific gates along the GI tract, which would in turn substantially increase the retention time of the carrier inside the GI tract and achieve a prolonged delivery regime. They introduced two potential spots for this scheme: The cavity right before the anus and the pyloric sphincter (the end part of the stomach) [160]. Extended retention behind the pylorus (~1.5 cm in diameter) was first implemented by producing expandable structures, where the expanded size would reach up to more than 2 cm in diameter [162]. However, the original versions of this device were causing serious problems, such as damaging fractured pieces or causing lumen obstruction, due to the non-degradable/non-dissociable nature of the materials used, necessitating surgery for removal [163].

In a comprehensive study, Zhang et al. built an elastic foldable O-ring made of various hydrogels [164]. They controlled the degradation rate and the mechanical properties of the structure by modifying the chemical composition and adjusting the ratio of the hydrogels, finally encapsulating it in a degradable capsule. The capsule dissolved in the stomach, leaving the opened O-ring in the stomach cavity. The drug-loaded segments of the ring degraded gradually and released the drug over several days [164]. By controlling the degradation rate of the samples, one can tune the release pattern of the samples. There might be two concerns associated with this new design: (1) If the ring stays in the stomach, it may block/affect the passage of the digested food through the GI tract. (2) If such structures reside in the GI tract for a long time, they may cause or help spread adverse effects. As for the first issue, no negative effect on the stooling pattern was observed in the animals that were experimented on in the study. Regarding the second concern (the device staying in the stomach for a long time), this was compared with indigestible food masses trapped inside the GI tract, which do not cause serious problems unless they grow very large. Although there have been answers to these concerns, the issue of how to remove the device from the pylorus in the case of toxicity or adverse reaction by the drug being released needs to be addressed. Also, the possibility that the ring accidentally passes by the pylorus and enters the intestine lumen remains [160].

### 3.3. Solvent-Free Microencapsulation

#### 3.3.1. Multiemulsion Systems

MPs have been the main candidates for oral microencapsulation systems [96,165,166,167]. MPs can be classified into two main categories: (1) Solid MPs, which are solid polymeric MPs with drug molecules dispersed in their matrix, and (2) hollow MPs, which are polymeric shells with hollow interior spaces that accommodate the delivered drug molecules [168,169]. In most of the protocols developed for the fabrication of MPs, drug molecules are directly involved in the fabrication process of the carrier, leading to direct contact between the drug molecules and the harsh organic solvents, raising concerns over drug denaturation, especially for biopharmaceuticals [170]. As such, the development of solvent-free microencapsulation technologies is of primary importance in drug delivery applications.

Multiemulsion systems (W/O/W and O/W/O) were originally developed to minimize contact between the drug and solvents, however, the traditional versions of these systems were difficult to make, control and stabilize, additionally being inefficient in terms of throughput [171,172,173,174,175,176,177]. While these systems minimized the contact between the drug molecules and organic solvents, they were not able to completely eliminate it due to partial contact at the O/W phase interface. For a wide range of applications of the emulsion technology, it is important to fully characterize and address the issues of drug denaturation along the fabrication process of the carriers, such as shear stresses caused by mechanical agitation or sonication and local temperature increases due to sonication. 

Microfluidic devices were successful in addressing a number of fundamental problems associated with the emulsion technique: highly uniform MPs with very small polydispersity index and considerable stability in liquid state emulsion [178,179]. Also, making multi-channel devices with various geometries is more straightforward and significantly more efficient in terms of minimizing O/W phase contact. Furthermore, there is no need for sonication or mechanical agitation to stabilize the emulsion made by microfluidic devices, resolving various problems associated with traditional emulsification techniques [180]. Due to these advantages, this new approach has attracted considerable attention for cell encapsulation/delivery. For example, pancreatic islet cells, as one of the most significant treatments for type 1 diabetes, can be successfully encapsulated and delivered using microfluidic devices [181]. The size, morphology and loads of the carriers can be controlled by regulating the flow rate of the phases (flow rate of the continuous phase and infusion rate of the dispersed phase), the geometry of the channels and nozzles and the concentrations of the emulsifiers [182]. Although microfluidic devices contributed to advance the emulsion technology, there are still problems with these systems, including the clogging of channels, attachment of oil phase/polymer fragments to the channel walls and also O/W contact at the interface [183,184]. As the main challenge against the widespread application of these systems is their low throughput, this prevents them from being scaled up to commercial extents and entering the pharmaceutical industry for mass production.

#### 3.3.2. Pored and Hollow Microencapsulation Systems

Another strategy for solvent-free microencapsulation is to employ separate processes for MP fabrication and drug encapsulation. That is, the drug can be loaded into the MPs in their favorable environmental conditions after the MP fabrication is complete, minimizing the possibility of drug denaturation due to the formation of MPs in the presence of the drug [185]. For this aim, several fabrication methods were previously developed, almost all of which were based on the same idea: coating solid spheres as templates with the desirable polymer material [186,187,188]. The templates (solid cores) were subsequently removed from the inside through either calcination or etching, leaving hollow polymeric spheres behind as the final product. These methods could hardly be successful due to the complications associated with diffusional material flow through the solid state polymeric shells. Since the drug can be loaded only by soaking the particles in a concentrated solution of drug, this method does not yield satisfactory levels of loading efficiency, limiting its general application [186,189,190].

Hyuk Im et al. proposed an idea to fabricate hollow polymeric microspheres with single surface pores using a solvent evaporation method [148]. Swollen solid polystyrene particles in organic solvents were plunged in liquid nitrogen, followed by slow evaporation of the solvent. The solvent would diffuse into the particles throughout the incubation step and would expand due to freezing, creating a hollow interior space inside the particles. The evaporation step would also generate surface pores, allowing for encapsulation of the cargo. It was demonstrated that the surface pores could then be closed through the thermal treatment of the particles above their glass transition temperature. 

**Figure 4:**
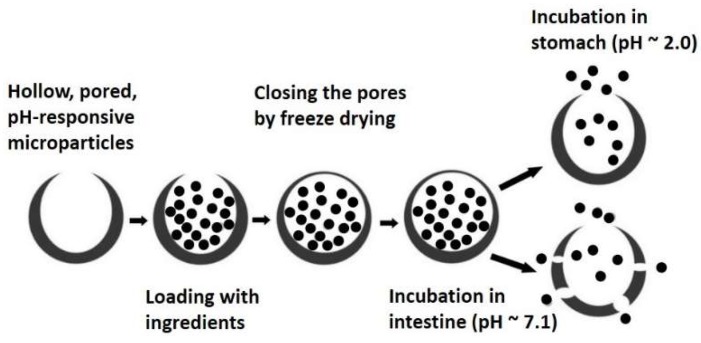
The schematic sequence of polymeric pored microencapsulation/release behavior.

More recently, a simplified method of making single pored-MPs was reported by Kumar et al. based on ultrasonic O/W emulsification [185]. The obtained macropores could be used for the direct loading of the drugs in their favorable conditions by applying vacuum cycles, and then closed to protect the drug during transition inside the stomach (Figure 4) [185]. In this method, the surface pores were sealed through freeze drying, thus eliminating the concern over the possible thermal denaturation of the biopharmaceuticals during the heat treatment of the MPs. It has been proposed that the pore closure mechanism is due to polymer-polymer interactions, followed by the removal of water. This new design was later modified to make larger MPs with larger surface pores, suitable for the delivery of large biomolecules and cells. 

### 3.4. Co-Delivery Systems

Although the co-delivery of drugs is not one of the core challenges associated with delivery systems, the administration of different drugs at the same time can play crucial roles in treatment efficacy in many cases. Delivering multiple drugs to target different sites at the same time can significantly reduce treatment time and the risk of failure [191]. Chemotherapy provides an example of co-delivery and its importance [191,192,193,194]. As another example, insulin delivery systems may cause a drop in the release of insulin due to frequent administration of the drug. The co-delivery of insulin and cyclic adenosine monophosphate (cAMP) was proposed to enhance the secretion of further insulin by activating the Ca^2+^ channels in beta cells of the pancreas [195]. It is thus of importance to develop co-delivery systems for the encapsulation and delivery of multi-target drugs. 

A straightforward solution for co-delivery is the simultaneous administration of different drugs in separate delivery carriers [196]. Another solution is the concurrent encapsulation of different drugs in micelles, liposomes or MPs. The implementation of this idea dates back to 2011, when an anticancer drug (MEK inhibitor PD0325901) and a therapeutic gene (Mcl1-specific siRNA (siMcl1)) were concurrently loaded into *N*′,*N*″-dioleylglutamide-containing cationic liposomes [197]. This new co-delivery strategy exhibited significantly enhanced anticancer activity both in vitro and in vivo. Cao et al. also simultaneously incorporated adenovirus encoding for murine interleukin-12 (Ad5) and paclitaxel (PTX) into anionic liposomes and performed in vitro/in vivo analyses, confirming that their co-delivery system (AL/Ad5/PTX) is an effective platform for treating melanoma [198]. 

**Figure 5:**
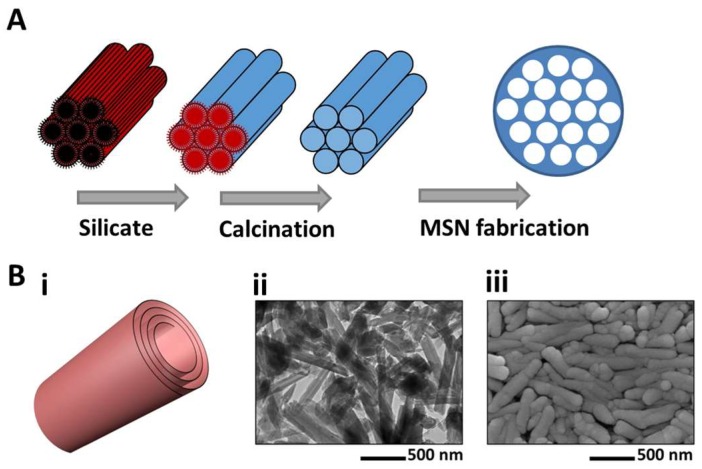
Oral delivery devices based on silica. (**A**) Fabrication of mesoporous silica nanoparticles (MSNs) (adapted with permission from [202]), (**B**) halloysite nanotube (HNT) microstructure ((**i**) schematic representation of a HNT, (**ii**) transmission electron microscopy (TEM) image and (**iii**) scanning electron microscopy (SEM) image).

Oral carriers based on SiO_2_ are also one of the most commonly used delivery vehicles, especially for co-delivery purposes [192,194,199]. Mesoporous silica nanoparticles (MSNs) and halloysite nanotubes (HNTs) are the principal examples of SiO_2_ structures investigated for such applications (Figure 5). HNTs are naturally forming structures, available in sufficient amounts in North America, China and New Zealand, making them a valuable candidate material in the pharmaceutical industry by meeting the requirements for scaling up and commercialization. As opposed to HNTs, MSNs require a time-consuming and expensive manufacturing process, although they have attracted significantly more attention as drug carriers compared to HNTs, mostly due to their larger interior space and higher loading capacity [200]. MSNs are nanoparticles made of silica with high chemical stability, a porous structure and the ability for surface modification/decoration [199,201]. Contrary to naturally forming HNTs, MSNs are artificially fabricated using a sol-gel method through the use of surfactants (Figure 5A). The idea of the addition of surfactants was first proposed by Kresge et al. and then modified by Inagaki et al. [202,203]. Concisely, hexagonal arrays of surfactant molecules are formed as micelles in a continuous aqueous phase, with hydrophobic segments in the center and hydrophilic fragments on the surface. Aluminosilicate is added afterwards to be adsorbed on the surface of the micelles and form the inorganic walls of the nanoparticles, and the organic core is finally extracted through thermal treatment. Co-delivery using MSNs has been investigated extensively by many researchers for the purpose of simultaneously delivering multiple drugs, and at the same time to minimize the multidrug resistance risk and lower the failure risk of the treatment. As an example, Chen et al. designed a co-delivery system for the simultaneous administration of siRNA and DOX, considerably modified the toxic effects of DOX (by ~132 times) [191].

HNTs are known as nonhazardous, non-degradable and biocompatible materials with no negative effects on different human cell lines, such as dermal fibroblasts and epithelial cells, making them favorable carriers for oral delivery, dermal treatments and cosmetics [191,204,205,206]. Notably, HNTs may not be suitable candidates for transdermal injections due to their size (0.4 to 1.5 μm long, which is slightly larger than the optimal length for biosafety, 1 μm). HNTs are spiral sheets of aluminosilicate kaolin, rolled 15–20 times over a single axis (15 nm inner diameter and 40–60 nm outer diameter) and have attracted attention as drug carriers, mainly for oral administration, however (Figure 5B) [200]. The inner and exterior sides of the lumen walls are made of alumina and silica, respectively. This double-layer structure enables the simultaneous loading of oppositely charged biomolecules. These are molecules with a negative zeta potential inside the lumen and those with a positive charge on the outer surface [207]. The outer negative charge can disperse particles in organic/aqueous environments and allow for further functionalization. The inner diameter of the lumen is also large enough to encapsulate both small drug molecules and larger macromolecules such as proteins [208]. 

**Figure 6:**
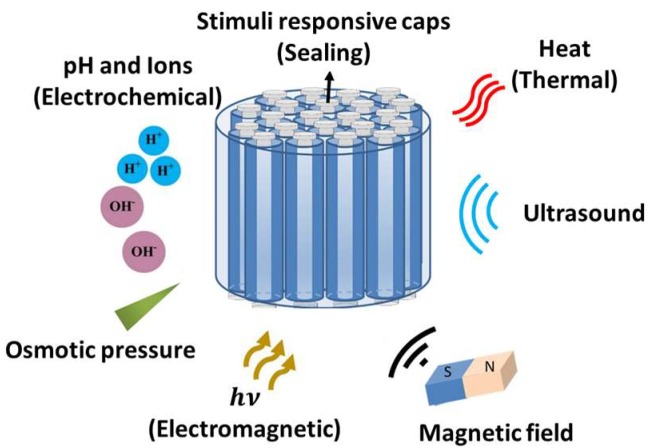
Different types of sealing strategies used for stimuli-responsive MSNs.

To apply HNTs and MSNs to drug delivery systems, it is required to seal their pore gates using a third material, known as a cap or gate keeper. Stimuli-responsive hydrogels have been mainly investigated as sealing materials to confer target-specificity to MSNs (Figure 6) [209,210,211,212,213]. Different types of stimuli (i.e., magnetic, light, thermal and pH) have been employed to date for MSN systems. Stimuli-responsivity in this microencapsulation scheme can be implemented through two main approaches: (1) Cleavable covalent bonding/crosslinking between the carrier and the drug in response to stimuli, such as the cleavable bonding at pH values below the plasma pH, and (2) functionalization of the surface or coating of the channels, which can switch conformation based on the surrounding properties and stimuli-responsive caps [192]. As an example, doxorubicin was conjugated to the interior walls of the channels in MSNs through pH-sensitive hydrazine bonds, which can prevent any untimely release of the drug. That is, upon being taken up into the cell through endocytosis, the acidic conditions of the endosomal/lysosomal environment can trigger the release of drugs due to the protonation of the bonding [214]. Another example includes light-responsive MSNs, made by Mekaru et al. using photoactivated azobenzene, which triggered release upon excitation by an external source of light [215]. These light-responsive MSNs are among the most common carriers used for cancer therapies. Magnetic-responsive MSNs later replaced them for the same purpose, since light could not penetrate deep enough into the tissue, while magnetic fields could address any kind of tissue at any desirable depth. Iron oxide was one of the best options for inducing magnetic-responsivity in MSNs [216,217]. Although these systems were not specifically targeted for oral drug delivery, they demonstrate the versatile stimuli-responsive properties that can embedded into these materials, which would be advantageous to oral drug carriers.

It should be noted that HNTs have attracted less attention than MSNs, although they are more readily available. HNTs would mainly be useful for applications requiring a small drug dosage due to their limited loading efficiency. The inner lumen usually shows about an 8–12% loading capacity, which can be increased to 30–40% by etching the HNTs in mild acidic environments [200]. Furthermore, MSNs structures can be modified to provide better control over the release pattern due to their tunable inner geometry.

### 3.5. Scaling up and Throughput

The technical challenges discussed so far mainly concern health-related issues. The throughput of the system, however, is the major challenge associated with the commercial implementation of the discussed technology. In general, almost all microencapsulation systems and protocols developed for the fabrication of microcarriers at a lab-scale suffer from low throughput and difficulty in scaling up. 

Among the microencapsulation approaches under current investigation, spray drying generally shows the highest throughput, fastest production rate and greatest ease of operation [218]. However, the direct contact between the drug molecules (genes, proteins, vaccine, biopharmaceuticals) and the organic solvents can readily denature/deactivate the drugs. In addition, spraying/dispersing the drug molecules in hot chambers may easily lead to thermal denaturation, and not all kinds of drugs may have satisfactory solubility in volatile organic solvents [219]. As a result, there has been a constant need to develop a universal, scalable drug delivery system without modification of the drugs.

Despite the many advantages emulsion microencapsulation techniques provide, their general application to drug delivery systems faces major challenges to be overcome. These challenges relate to problems including yield, polydispersity and sonochemistry. As discussed previously, microfluidic systems could properly address most of the problems associated with traditional emulsification methods at the expense of yielding a lower output [220]. Regardless of its geometry, a single microfluidic channel can yield a throughput of emulsion droplets in the range of 0.1–10 mL/h with a very narrow size distribution (<5%), while the pharmaceutical industry is looking for much higher values (above 1 L/h) [221]. Traditional emulsification techniques may show a much higher yield (100–20,000 L/h) at the expense of the quality of the droplets and polydispersity range [222]. Ofner et al. proposed a multi-parallel channel microfluidic device to improve the throughput of microfluidic systems, reporting promising values that would satisfy commercial needs [219]. Tendulkar et al. implemented the same idea of the parallelization of several channels in a more complex design of microfluidic devices for the encapsulation of islet cells to treat type 1 diabetes [223]. They included an air jet supply to aid the detachment of the droplets from the T-junction nozzles and increase the throughput of the system, reporting a 8–64-fold increase in the production rate. Although multi-channel designs look to be a promising solution for increasing the throughput of MP fabrication, when comparing with the commercially required flow rates with bench-scale infusion rates from a single channel, about 1000 channels may be needed for attaining a satisfactory level of throughput. Nisisako et al. [224] and Conchouso et al. [225], in separate studies, proposed 3D circular arrays of channels to both minimize the flow rate distribution among the channels and increase the throughput of the system. These complex microfluidic devices can be made of metal, polymer or glass, and 3D printing, acid etching and computer numerical control CNC engraving are common practices for forming the channels [219,221,223,224,225]. It should be noted that systems’ output in microfluidic devices is usually reported as the volume produced per minute [220,226], which may not be the ideal way for reporting the throughput of the system. In this regard, it would be necessary to better evaluate the performance of the system in terms of what fraction of the volume reported really represents the MPs produced, and what fraction is due to the contribution of other components of the system (e.g., continuous phase or random polymer fragments).

## 4. Conclusions

Although oral delivery is considered to be the most promising administration route due to its specific advantages, it faces substantial challenges that need to be addressed before oral systems can be commercially available for the delivery of biopharmaceuticals. Fabrication protocols of the carriers should adequately avoid any destructive effect (such as contact with organic solvents, shear stresses and local temperature increases) on the drug molecules, especially for biopharmaceutical encapsulation/delivery. Concurrently, the delivery material, design, size and polydispersity must be accurately controlled, due to their significant influence on treatment efficacy. Oral carriers deal with various biological barriers (the lumen, mucus and tissue of the GI tract) to successfully deliver drugs. Sustained delivery, solvent-free microencapsulation and co-delivery have also been major focuses in oral delivery studies. Nonetheless, the most significant issue against the commercialization of the oral systems is their low throughput. This review introduced the most promising solutions recently proposed for each barrier, which point to a positive progress in the field of oral drug delivery. In addition, the recent solutions proposed for scaling up the microencapsulation techniques, the parallelization of the microfluidic channels, are both significantly increasing the systems yield and addressing the other challenges as well. Due to the broadness, as well as the variety of the challenges against the oral drug delivery systems, we expect several different focuses for future research in this field. Considering the recent achievements in the case of technological challenges, we believe that future research in this field will mainly target biological barriers, especially the barriers associated with tissues. The main advantages of oral delivery systems, include sustained delivery, interaction with mucus and the capability for solid formulations that preserve pharmaceuticals, still making this the most attractive administration route for pharmaceuticals. 

## Figures and Tables

**Table 1:** Characteristics of different segments of the human gastrointestinal (GI) tract.

Segment	pH	Length (cm)	Mean Diameter (cm)	Mucus Average Thickness (µm)	Mucus Turnover (hour)
Stomach	0.8–5 [45]	20 [45]	NA	245 ± 200 [38]	24–48 [46]
Duodenum	~7 [38]	17–56 [47]	4 [48]	15.5 [45]	24–48 [46]
Jejunum	≥ 7 [38]	280–1000 [47]	2–2.5 [48]	15.5 [45]	
Ileum	≥ 7 [38]		3 [48]	15.5 [45]	
Colon	7–8 [38]	80–313 [47]	4–4.8 [48]	135 ± 25 [38]	24–48 [46]
